# Comparison of cytokine and gene activities in tissue and blood samples of patients with celiac disease 

**Published:** 2019

**Authors:** Ensieh KhalKhal, Zahra Razzaghi, Hakimeh Zali, Ayad Bahadorimonfared, Majid Iranshahi, Mohammad Rostami-Nejad

**Affiliations:** 1 *Proteomics Research Center, Faculty of Paramedical Sciences, Shahid Beheshti University of Medical Sciences, Tehran, Iran*; 2 *Laser Application in Medical Sciences Research Center, Shahid Beheshti University of Medical Sciences, Tehran, Iran.*; 3 *Proteomics Research Center, School of Advanced Technologies in Medicine, Shahid Beheshti University of Medical Sciences, Tehran, Iran.*; 4 *Department of Health & Community Medicine, Faculty of Medicine, Shahid Beheshti University of Medical Sciences, Tehran, Iran*; 5 *Gastroenterology and Liver Diseases Research Center, Research Institute for Gastroenterology and Liver Diseases, Shahid Beheshti University of Medical Sciences, Tehran, Iran *

**Keywords:** Celiac disease, PBMs, Gene expression, Intestine biopsy

## Abstract

**Aim:**

The aim of this study is to explore the expression of genes associated to celiac disease (CD) in the target tissue and peripheral blood monocytes (PBMC) or serum to introduce possible potential biomarkers.

**Background:**

Celiac disease (CD) is an autoimmune disease induced by gluten ingestion in genetically predisposed individuals. Despite technological progress, small intestine biopsy is still the gold standard for diagnosis of CD.

**Methods:**

CD data were collected from public databases (proteomics and microarray-based techniques documents). Differentially expressed genes (DEGs) in PBMC or serum as well as small intestinal biopsies from celiac patients compared to normal were collected and analyzed to introduce common individuals. Gene ontology was done to identify the involved biological terms.

**Results:**

Among 598 CD genes in biopsies and 260 genes in PBMC or serum, 32 common genes with a similar expression pattern in both sources were identified. A total of 48 biological terms were introduced which were involved in the CD via the determined DEGs. “Cytokine activity” was the most expanded one of the biological terms.

**Conclusion:**

In this analysis, it was concluded that 32 potential biomarkers of CD can be assessed by complementary research to introduce effective and available biomarkers in biopsy and blood.

## Introduction

 Celiac disease (CD) with a global prevalence of 1% is a chronic autoimmune disorder in genetically susceptible individuals which is triggered by wheat gluten. It is a multifactorial disease induced by genetic (HLA-DQ2 or HLA-DQ8) and environmental factors (gluten) (1, 2). Inflammation and villous atrophy of the small intestine are caused by undesirable immunological reactions. Villous atrophy can result in malabsorption of vitamins and nutrients (3). The CD patients may experience osteoporosis, iron deficiency, and bone disease due to nutritional deficiency (4). A permanent gluten-free diet (GFD) is the only accepted therapy for CD with most individuals responding to it (5). CD is associated with some diseases including insulin-dependent diabetes mellitus (6-8), non-alcoholic fatty liver disease, autoimmune hepatitis (9), hypothyroidism (6, 8, 10), Sjögren syndrome (7), Hashimoto thyroiditis, psoriasis (7, 8), inflammatory bowel diseases, asthma, arthritis, migraine, bile stones, and menstrual abnormalities (8). CD diagnosis is based on specific serological tests and the presence of a characteristic enteropathy in an intestinal biopsy (11, 12). Genetic and non-genetic factors such as epigenetics and the environmental factors cause most human diseases which share common genetic profiles. The available information for each disease constitutes a challenge in identification of disease-specific mechanisms. Hence, the combination of information from various databases and multi-source data such as proteomics, genomics, metabolomics, and microarray-based techniques can be crucial in understanding the pathophysiology and the common biological basis among different diseases. Achievement of a deeper understanding of the molecular mechanisms of diseases can be useful for detection of many unknown molecular aspects, development of more precise treatments for diseases as well as for biomarker discovery. To this end, we have integrated data to detect the key genes involved in the CD; thus, more effective and early diagnosis could be made in patients and to identify healthy at-risk individuals (13-25).

**Table 1:** Biological processes (BP) and molecular function (MF) related to the 32 common DEGs in both CD intestine and blood samples

R	Gene name	No. of genes	Biological process and molecular function	NO. of BP and MF
1	APOA1	1	apolipoprotein A-I receptor binding, beta-amyloid binding, chemorepellent activity, high-density lipoprotein particle binding, phosphatidylcholine binding, phosphatidylcholine-sterol O-acyltransferase activator activity, phospholipid transporter activity	6
2	ARG2	1	arginase activity	1
3	CXCL10	1	cAMP-dependent protein kinase regulator activity	1
4	IL2,CHI3L1	2	carbohydrate binding	1
5	PCK1	1	carboxylic acid binding, GDP binding, manganese ion binding, phosphoenolpyruvate carboxykinase (GTP) activity	2
6	CCR9,CCR3	2	C-C chemokine receptor activity	1
7	CLU	1	chaperone binding, misfolded protein binding	1
8	CXCL8,CXCL10,CXCL11	3	chemokine activity	1
9	CCR9,CCR3	2	chemokine receptor activity	1
10	CHI3L1	1	chitinase activity, chitin binding	2
11	APOC3,APOA1	2	cholesterol binding, high-density lipoprotein particle receptor binding, lipase inhibitor activity	3
12	APOB,APOA1	2	cholesterol transporter activity	1
13	CXCL10,CXCL11	2	CXCR3 chemokine receptor binding	1
14	IL21,IL12B,IL10,IL15,IL18,IL2,IL4, IL6,IFNG,IL23A,IL17A	11	cytokine activity	1
15	TNFRSF9	1	cytokine binding,	1
16	IL12B	1	cytokine receptor activity, interleukin-12 alpha subunit binding, interleukin-12 receptor binding	3
17	IL21,IL15,IL17A	3	cytokine receptor binding	1
18	STAT1	1	double-stranded DNA binding, nuclear hormone receptor binding, RNA polymerase II core promoter sequence-specific DNA binding, transcription factor activity, RNA polymerase II core promoter sequence-specific, tumor necrosis factor receptor binding	6
19	APOC3	1	enzyme regulator activity	1
20	CHI3L1	1	extracellular matrix structural constituent	1
21	APOH	1	glycoprotein binding, lipoprotein lipase activator activity	2
22	IL2	1	glycosphingolipid binding, kappa-type opioid receptor binding, kinase activator activity	3
23	RGS1	1	G-protein alpha-subunit binding	1
24	IL12B,IL10,IL2,IL4,IL6	5	growth factor activity	1
25	RGS1,TAGAP	2	GTPase activator activity	1
26	MPO,APOH,APOB,CXCL10,CXCL11	5	heparin binding	1
27	TNFAIP3,APOH,IL12B,STAT1,APOA1	5	identical protein binding	1
28	IFNG	1	interferon-gamma receptor binding	1
29	IL10	1	interleukin-10 receptor binding	1
30	IL12B,IL23A	2	interleukin-23 receptor binding	1
31	IL21,IL2	2	interleukin-2 receptor binding	1
32	IL4	1	interleukin-4 receptor binding	1
33	IL6	1	interleukin-6 receptor binding	1
34	CXCL8	1	interleukin-8 receptor binding	1
35	TNFAIP3	1	K63-linked polyubiquitin binding, kinase binding, protein self-association, thiol-dependent ubiquitin-specific protease activity, ubiquitin binding	4
36	APOB	1	lipase binding, low-density lipoprotein particle receptor binding	2
37	MPO	1	peroxidase activity	1
38	APOH,APOB,APOC3,APOA1	4	phospholipid binding	1
39	TNFAIP3,LCN2	3	protease binding	1
40	RBP4,RBP2	2	retinol binding	1
41	REL	1	RNA polymerase II distal enhancer sequence-specific DNA binding, transcriptional activator activity, RNA polymerase II distal enhancer sequence-specific binding	3
42	SH2B3	1	signaling adaptor activity	1
43	SH2B3,STAT1	2	signal transducer activity	1
44	LCN2	1	small molecule binding	1
45	RBP2,LCN2	2	transporter activity	1
46	TNFRSF14,TNFRSF9	2	tumor necrosis factor-activated receptor activity	1
47	CLU,TNFRSF14	2	ubiquitin protein ligase binding	1
48	TNFRSF14	1	virus receptor activity	1

**Table 2:** A list of genes that are upregulated or downregulated in the small intestinal and in PBMC or serum of celiac patients

	Up in both source		Down in both source	down in biopsy up in blood	up in biopsy down blood
1	ANXA3		APOH	APOA1	IL10
2	ARG2		LPP	APOB	IL6
3	CCR3		RBP	APOC3	INPP4A
4	CCR6			CHI3L1	RBP4
5	CCR9			IL18	RGS1
6	CLU			JAM3	SH2B3
7	CXCL10			KIAA1109	STAT1
8	CXCL11			PCK1	TNFRSF14
9	GUCY1B3				
10	IFNG				
11	IL12B				
12	IL15				
13	IL17A				
14	IL2				
15	IL21				
16	IL23A				
17	IL4				
18	IL6				
19	IL8				
20	KRT19				
21	LCN2				
22	MPO				
23	OLFM4				
24	pSTAT1				
25	REL				
26	SH2B3				
27	TAGAP				
28	TBX21				
29	TNFAIP3				
30	TNFRSF9				

The aim of this study is to explore the common differentially expressed genes in the intestine and peripheral blood monocytes (PBMC) or serum of CD patients to introduce readily available biomarker candidates for CD. 

## Methods

All data including proteomics and microarray-based documents about CD were collected from public databases and gene expression databases. CD related data were extracted from NCBI (https://www.ncbi.nlm.nih.gov/pubmed) which were published by March 2019. We used “Celiac Disease”, “duodenum or biopsy”, “proteomic”, “microarray or RT-PCR”, and “PBMC” as the main keywords for searching in NCBI and Google Scholar to find the genes or proteins that are up or downregulated in CD comparison to healthy control.

**Figure 1:**
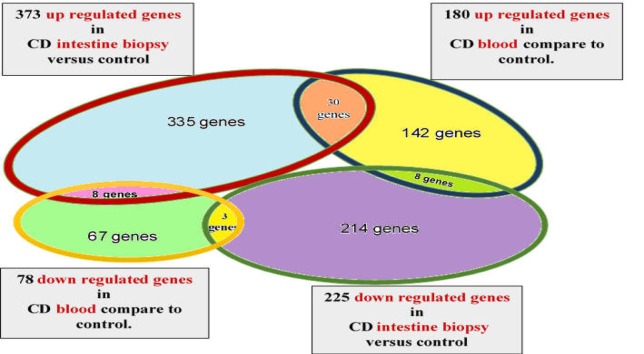
The number of common and differentially expressed genes in both of CD blood and tissue

The “Celiac Disease”, “celiac disease + duodenum or biopsy + proteomic”, “celiac disease + PBMC + proteomic”, “celiac disease + duodenum or biopsy + microarray or RT-PCR”, “celiac disease + PBMC + microarray or RT-PCR” parent terms were used. The common DEGs between intestine biopsy and blood samples were identified. Also, the DEGs with a similar expression pattern in both samples were enriched by Reactome application of Cytoscape software for biological processes and molecular function. 

## Results

Table 1 presents the genes that display differential expression in PBMC or serum as well as CD small intestinal biopsies, as compared with normal small intestinal biopsies and PBMC or serum. In order to integrate data, we combine the up- and downregulated genes involved in celiac patients compared to healthy controls. There are 373 upregulated and 225 downregulated genes in the intestine tissue of celiacs versus controls. Also, 180 upregulated and 78 downregulated genes of different gene expression profiles were identified in the peripheral blood mononuclear cells (PBMCs) or serum of celiac patients versus controls (19, 26-38). 

There are 49 common differentially expressed genes including 33 genes with a similar pattern of regulation and 16 ones with an opposite outline in both CD small intestinal biopsies and blood (Figure 1). Table 2 reports different gene expression profiles in peripheral PBMCs or serum, as well as tissue of celiac patients.

 ANXA3, ARG2, CCR3, CCR6, CCR9, CLU, CXCL10, CXCL11, GUCY1B3, IFNG, IL12B, IL15, IL17A, IL2, IL21, IL23A, IL4, IL6, IL8, KRT19, LCN2, MPO, OLFM4, STAT1, REL, TAGAP, TBX21, TNFAIP3, and TNFRSF9 are upregulated while APOH, LPP, and RBP are downregulated in both samples of intestine and blood. APOA1, APOB, APOC3, CHI3L1, IL18, JAM3, KIAA1109, and PCK1 are downregulated in the intestine and upregulated in blood samples. IL10, IL6, INPP4A, RGS1, SH2B3, STAT1, and TNFRSF14 are the genes that are upregulated in the intestine biopsy and downregulated in the blood samples. The biological processes and molecular functions related to the 33 common DEGs with a similar expression change in both samples are shown in Table 1.

## Discussion

In the present study, we tried to integrate CD related data (differentially expressed genes). Integration and analysis of these data is useful to uncover critical molecular mechanisms of CD and facilitate diagnostic biomarker discovery. In order to mix data, we combined up and down-regulated genes involved in celiac patients as compared to healthy controls. Amongst them, 47 CD genes had altered expression in both CD small intestinal biopsies and blood. The genes with a common expression pattern in blood and intestinal tissue were thirty-two while the other 15 were expressed in an opposite way. 

As mentioned earlier, APOH, APOB, APOA1, APOA4, APOC2, and APOC3 are downregulated in the CD intestinal, while only Beta-2-glycoprotein 1 (APOH) among them is downregulated in both sources. It is shown that Intestinal apolipoproteins play a critical role in lipoprotein metabolism. Some of them are synthesized with chylomicrons in the mucosa and secreted into the intestinal lymph. This seems to be important for the secretion of chylomicrons and to stay with chylomicrons during their lipolysis in plasma (39). Because of villous atrophy and apolipoprotein reduction, fat and cholesterol malabsorption in CD patients is often increased causing hypocholesterolemia. Consequently, the absorption efficiency of cholesterol is impaired (40). The APOH serum protein binds to phospholipids, heparin, and dextran sulfate and prevents intracellular coagulation cascade activation by binding to phospholipids on the surface of damaged cells. It binds to the membrane of T cell, and supports its function as a marker for membrane flip flop. In addition, it is involved in inflammatory mediated process of cell death (41) as well as activation of endothelial cells and apoptotic mechanisms (42). It is a part of the group of acute phase proteins (43) whose expression is sometimes upregulated and sometimes downregulated, reflecting its pleiotropic functions such as APOH reduction in CSF during cerebral malaria (44) and rises in several infectious diseases (45). It can be one of the major elements of the first line of the innate immune response regulating homeostasis. 

The other common downregulated gene in both samples is Retinol-binding protein (RBP). It is known as an adipocyte-secreted protein which transports retinol in the blood from its storage site in the liver to the epithelial tissues. RBP is a retinol transporter protein present in the small intestinal epithelium. It plays an important role in the uptake and intracellular metabolism of vitamin A. It also modulates the rate of retinoic acid supply to the endometrial cell nuclei during the menstrual cycle (46, 47). The RBP plasma concentration is regulated by vitamin A status, where in vitamin A deficiency, RBP molecules are not secreted from the liver and decline in plasma (46). Retinol is a vitamin necessary for growth, reproduction, differentiation of epithelial tissues, and vision. Malabsorption is frequently encountered in CD-patients because of villous atrophy and crypt hyperplasia in the duodenum and jejunum; thus, vitamin and mineral deficiencies e.g. vitamin A deficiency is reported in CD-patients. Vitamin A deficiency blocks secretion of the binding protein post transnationally and results in defective delivery and supply to the epidermal cells (48). 

Lipoma-preferred partner (LPP), the third downregulated gene is involved in cell motility and cell adhesion which is crucial to maintaining the barrier integrity of the small intestine. Therefore, it might play a role in the pathogenesis of CD (49) 

A total of 29 of these genes have had higher expression levels in both sources compared to controls. They are directly or indirectly involved in the inflammation process, and were significantly up-regulated in the epithelial cells of CD patients relative to controls. They have a specific role in the gluten-induced immune response (see Table 1).

Both c-REL ( roto-oncogene c-REL) and TNFAIP3 (Tumor necrosis factor alpha-induced protein 3) are involved in the regulation of the nuclear factor kappa β (NF-kβ) inflammatory signaling pathway in the pathology of CD, and are the key mediators in this nuclear activating complex (50). NF-kβ is a transcription complex which plays a key role in regulating the cellular immune response to stimuli. In various inflammatory disorders, including asthma, arthritis, and inflammatory bowel disease, NF-kβ is activated whose pathway may play an independent role in the innate mechanisms of disease development (51). c-REL is a member of NF- kβ transcription factor family and has high levels of expression in B and T cells, with many target genes involved in lymphoid cell growth and survival. It plays a major role in promoting immune and inflammatory responses through producing pro-inflammatory cytokines. It has been associated with autoimmune diseases such as arthritis (52), psoriasis (37), and CD (50). REL expression increases in a variety of B and T cell malignancies as well as in other cancer types. Thus, agents that modulate REL activity may have therapeutic benefits for chronic inflammatory diseases and human cancers (53). TNFAIP3, also known as A20, has been associated with psoriasis (54), rheumatoid arthritis (55), type 1 diabetes mellitus (56), systemic lupus erythematosus (57), and CD (50). TNFAIP3 expression is up-regulated by NF-kB activation, and acts in a negative feedback to control NF-kβ-dependent gene expression. It blocks NF- kβ activation which mediates immune responses (58); thus, TNFAIP3 plays a key role in controlling inflammation in autoimmune diseases and is suggested as an attractive candidate for drug targeting (59). Also, TNFAIP3 is frequently inactivated in subsets of B-lineage lymphomas that are characterized by NF-kB hyper activation. Hence, it could be suggested as a novel tumor suppressor (60).

The inflammatory process involves infiltration of macrophages, B-lymphocytes, plasma cells and mainly T lymphocytes. T-cells can be divided into Th1 (T helper 1) or Th2 (T helper 2) subsets based on their cytokine secretion; IFN-ɣ and IL-2 are the main cytokines secreted by Th1 cells while IL-4 is secreted by the Th2 cells. Cytokine secretion from lymphocytes is higher among celiac patients as compared to controls. Elevated IFN-ɣ and IL-2 secretion has been detected in mucosal tissue lymphocytes and peripheral blood T lymphocytes (61-63). TBX21 (T-box transcription factor TBX21) and pSTAT1 are important transcription factors for the development of T helper type 1 (Th1) cells and control of Th1 cytokine expression plus IFNɣ. Its expression is also associated with IFNɣ expression. Th1 cells and natural killer play an important role for this gene in initiating Th1 linage development of Th-naive precursor cells (19, 64). 

RGS1 (Regulator of G-protein signaling 1) controls the homing of intraepithelial lymphocytes (IELs) which are less active in CD patients than in controls while being essential for producing the gluten induced epithelial damage. IELs play an important role in preventing unwanted and over-inflammatory reactions that can damage the intestinal epithelium. It is also able to prevent the entry and spread of pathogens thus preventing the development of immunity. IELs eliminate infected and damaged cells, as well as regulatory functions that contribute to epithelium healing and repairing. A hallmark of CD is deregulated activation of IELs. It is involved in epithelial cell destruction and the subsequent development of villous atrophy. 

The expression of SH2B3 (SH2B adapter protein 3) and RGS1 is lower in CD peripheral monocytes versus controls and higher in CD duodenal intestinal samples than in controls. The capacity of RGS1 to limit the egress of inflammatory and autoimmune cells could encourage the immunopathology of autoimmune disorders (65). SH2B3 is associated with many autoimmune and cardiovascular disorders, including type 1 diabetes mellitus, celiac disease, myocardial infarction, and hypertension. It is strongly expressed in the inflamed small intestines of CD patients. This alteration leads to modulation of intermolecular interactions and inhibition of cytokine responses. Its function is found in the innate immune response. SH2B3 negatively regulates lymphopoiesis and early hematopoiesis and reduces the Th-1 cell response in the context of CD etiology. Deficiency of SH2B3 leads to enhanced production of B-cells (66). 

PCK1 is an enzyme that regulates gluconeogenesis. At low glucose levels, it catalyzes the oxaloacetate (OAA) to phosphoenolpyruvate (PEP) while at high glucose levels, it catalyzes PEP to OAA. It has a metabolic function in diseases (67). KIAA1109 level is higher in CD blood monocytes and lower in CD intestinal tissue samples versus controls. KIAA1109 is expressed in many different tissues e.g. parathyroid, muscle, ear, eye, mammary gland, lymph nodes, and thymus in humans. It is also expressed in various diseases e.g. bladder carcinoma, chondrosarcoma, glioma, leukemia, lymphoma, non-neoplasia, and retinoblastoma tissues. In mammals, its function is regulating epithelial growth and differentiation, tumor development, as well as in inflammatory conditions affecting the gastrointestinal tract, type I diabetes, and rheumatoid arthritis (68-71).

Gen ontology analysis revealed more information about roles of DEGs in the onset and promotion of CD. The important biological term which appeared as a term with maximum DEGs involution is “cytokine activity”. There are 11 DEGs among the 32 introduce genes that are involved in this biological term. This finding is affected by the fact that immune system is the main dysregulated part in CD.

As an overview in CD, the genes involved in inflammatory processes are upregulated, while the genes in the cell adhesion or integrity of the intestinal barrier are downregulated (34).

Since blood is a more available source than intestinal biopsy, finding common DEGs in both samples is a useful task to introduce potential biomarkers. In this regard, about 5% of tissues DEGs were identified as blood DEGs. Most of these potential biomarkers were determined as immune system elements.
